# Developmental trajectories of suicide risk in college students: a three-year Latent Growth Mixed Model study

**DOI:** 10.3389/fpsyg.2025.1584446

**Published:** 2025-06-06

**Authors:** Liu Zhuojun, Liu Mian, Zhang Zhifang, Chen Zhuangyou

**Affiliations:** Mental Health Education and Counseling Center, Dongguan University of Technology, Dongguan, Guangdong, China

**Keywords:** suicide risk, college student, Latent Growth Mixed Model, developmental trajectories, risk factors

## Abstract

**Background:**

This study aimed to explore the developmental trajectories of suicide risk among college students and examine the influence of demographic, psychological, and social factors on these trajectories.

**Methods:**

A three-year follow-up study was conducted with 3,723 first-year college students from a university in Guangdong Province, China. Data were collected in October 2020, 2021, and 2022 using the Suicide Behaviors Questionnaire-Revised (SBQ-R), University Personality Inventory (UPI), and Self-rating Depression Scale (SDS). Latent Growth Mixed Modeling (LGMM) was employed to analyze the trajectories of suicide risk.

**Results:**

Three distinct trajectories were identified: a “slowly decreasing suicide risk group” (81.1%), a “slowly increasing suicide risk group” (15.7%), and a “rapidly increasing suicide risk group” (3.2%). Female gender, left-behind experience, history of suicide among close relatives or acquaintances, positive psychological symptoms, and depressive symptoms were significant risk factors for higher suicide risk trajectories (all *p* < 0.05).

**Discussion:**

The findings highlight significant heterogeneity in suicide risk trajectories among college students, emphasizing the need for targeted interventions based on individual risk profiles.

## 1 Introduction

Suicide is a leading cause of death globally, with an estimated 703,000 deaths annually, accounting for 1.3% of all deaths worldwide (World Health Organization, 2019). Among young people aged 15–29, suicide is the fourth leading cause of death (World Health Organization, 2019). College students, in particular, are at heightened risk for suicidal behavior due to academic pressures, social challenges, and mental health issues (Zhou et al., 2021). Suicide not only has devastating effects on individuals but also profoundly impacts families, schools, and society at large. In 2023, the notice of the “Special Action Plan for Comprehensively Strengthening and Improving Students’ Mental Health Work in the New Era (2023–2025)” jointly issued by 17 departments including the Ministry of Education clearly required improving psychological early warning and intervention, early detection of students’ serious mental health problems, monitoring and early warning of students’ self-harm, suicide, or injury-causing behaviors, and emphasized further promoting the application of scientific research results in students’ mental health monitoring, early warning, and intervention. With the intensification of social competition, college students are facing increasing pressures in academics, social interactions, and employment. Mental health problems are becoming more prominent, and suicide incidents occur from time to time. In the current social context, the increasing pressure on college students has led to a rise in mental health problems, making it particularly necessary to understand the developmental trajectories of suicide risk and related influencing factors.

While suicide is preventable, understanding its risk factors and developmental trajectories is crucial for effective prevention strategies. In terms of demographic factors, previous studies have found that gender and sexual orientation are influencing factors of suicide risk among college students. Females are more sensitive to emotional changes and may be more affected by stress, increasing their suicide risk. Females and sexual minorities are risk factors for the transition of suicide risk from low to high among college students with positive psychological symptoms during a one-year follow-up. Moreover, being heterosexual is a protective factor for the transition of suicide risk from high to low among such students. Being in a position of “outsider” or “minority” in the social environment may still cause long-term stress and a sense of threat (such as discrimination or bullying) among sexual minorities (Che and Jia, 2023; Chen et al., 2023; Xue et al., 2023). At the social level, college students with a history of suicide exposure and left-behind experience (before the age of 18, being separated from one or both parents for more than half a year) are more likely to face a higher risk of suicide due to factors such as a sense of belonging frustration, weak social support, imitation effects, and learned helplessness (Li et al., 2022; Wang et al., 2022; Sui, 2024; Qi et al., 2024; Zhu et al., 2024). Internalizing psychopathology, such as depressive symptoms and anxiety symptoms, is often closely associated with the risk of suicide behavior. These factors have a strong theoretical basis and practical significance in the study of suicide risk among college students (Altavini et al., 2023).

Previous research has extensively examined the prevalence (He et al., 2019), risk factors (Chen et al., 2023; Xue et al., 2024), and mechanisms (Lu, 2021) of suicide risk among college students. However, limited attention has been paid to the developmental trajectories of suicide risk and the heterogeneity among different risk groups. Suicide risk is not static; it evolves over time due to the interplay of multiple factors, including demographic, psychological, and social influences. Identifying distinct trajectories of suicide risk can help tailor interventions to specific subgroups of students.

Integrating the interpersonal theory of suicide and the stress-vulnerability model, this study employs Latent Growth Mixed Modeling (LGMM) to explore the developmental trajectories of suicide risk among college students over a three-year period. Based on the above background, the following hypotheses are proposed: H1: There is significant heterogeneity in the suicide behavior risk trajectories of college students, and at least three subgroups can be distinguished. H2: Female students, those with a left-behind experience, a history of suicide among close relatives or acquaintances, positive psychological symptoms, and depressive symptoms are more likely to belong to the risk-increasing trajectories. We aim to (1) identify distinct trajectories of suicide risk, (2) examine the impact of demographic, psychological, and social factors on these trajectories, and (3) provide evidence-based insights for targeted suicide prevention strategies. This study can provide practical guidance for colleges and universities to formulate scientific and effective suicide prevention and intervention measures, which is of great significance for protecting the mental health and safety of college students.

## 2 Methods

### 2.1 Participants and procedure

A total of 5,116 first-year college students from a university in Guangdong Province, China, were recruited using a cluster sampling method. Data were collected at three time points: October 2020, October 2021, and October 2022. We used the “academic year” as the unit of time measurement. The data collection time points were coded according to the academic years. The 2020–2021 academic year was coded as Time Point 1, the 2021–2022 academic year as Time Point 2, and the 2022–2023 academic year as Time Point 3. The study and life of college students are usually arranged in cycles of academic years. Using the academic year as the unit of time can better reflect the association between the changes in college students’ suicide risk over time and their actual life situations. Participants completed surveys assessing suicide risk, psychological symptoms, depressive symptoms, and written informed consent was acquired from all participants. After excluding incomplete or careless responses (such as a large number of consecutive identical options in the questionnaire, answering time being too short, e.g., less than 5 min, and logically inconsistent answers), 3,723 valid datasets were retained for analysis. There were 2,419 male students (accounting for 65%) and 1,304 female students (accounting for 35%). At the first measurement in 2020, the age scores of the surveyed subjects ranged from 15 to 26 years old, with a mean ± standard deviation of 18.33 ± 0.75. The number of students whose family residences were in rural areas, county towns, and cities were 1,158 (31.1%), 877 (26.2%), and 1,591 (42.8%) respectively. The number of students from poor families was 465 (12.5%). Chi-square tests confirmed no significant differences (all *p* > 0.05) in demographic or psychological variables between participants who completed all surveys and those who dropped out, indicating no structural loss of data.

In the actual research process, since our research was a questionnaire-based study and the questionnaire content mainly focused on common issues related to college students’ mental health, without involving invasive operations or collection of sensitive private information that might pose significant risks or harm to participants, we did not apply for approval from an ethics committee following the strict process at that time. However, we attached great importance to research ethics. Before data collection, the school psychological center used social media and video introductions to provide students with detailed information about the purpose, significance, and confidentiality principles of this survey, ensuring that students participated voluntarily after full understanding. Although we did not sign written informed consent forms, at the beginning of the survey, a special notification section was set up on the questionnaire page, clearly informing students that their participation in the survey was regarded as consent to participate in this study. Students could only enter the questionnaire answering interface after clicking the “Agree” button, thus ensuring students’ right to know and the right to choose independently.

### 2.2 Measures

#### 2.2.1 Demographic and sociological information

A self-compiled questionnaire was used to collect demographic and sociological data, including gender, left-behind status, only-child status, sexual orientation, and history of suicide among close relatives or acquaintances.

#### 2.2.2 University personality inventory (UPI)

The 64-item UPI evaluates general psychological symptoms (56 items), with additional lie-scale and auxiliary items. Responses are binary (0 = “no,” 1 = “yes”), with total scores ranging from 0 to 56 (higher scores indicate poorer mental health). Participants were classified as having “positive psychological symptoms” if they met one of four criteria: (1) total score ≥ 25, (2) endorsement of item 25 (“Do you often think about death?”), (3) ≥ 2 affirmative auxiliary items, or (4) explicit counseling requests. Cronbach’s *α* in this study was 0.901.

#### 2.2.3 Self-rating depression scale (SDS)

The SDS, validated in Chinese populations, includes 20 items rated on a 4-point Likert scale. Standard scores (raw score × 1.25) ≥ 53, ≥ 63, and ≥ 73 indicate mild, moderate, and severe depression, respectively. Weighted average scores across three time points were used. Cronbach’s *α* ranged from 0.838 to 0.864. (China Employment Training Technical Instruction Center, 2005).

#### 2.2.4 Suicide behaviors questionnaire-revised (SBQ-R)

The 4-item SBQ-R, adapted for Chinese students (Shi et al., 2021), assesses lifetime suicidality, past-year ideation frequency, threats, and future likelihood. Total scores range from 3 to 18, with ≥ 7 indicating high risk. Cronbach’s α ranged from 0.713 to 0.757.

### 2.3 Quality control

Prior to data collection, the school psychological center disseminated information about the psychological census through social media and video introductions. Common student questions were addressed, and the purpose and confidentiality of the assessment were emphasized. During data collection, trained examiners from the psychological counseling stations administered the surveys in class units using the Zhiwei system. Electronic questionnaires were distributed, completed, and collected at designated times and locations to ensure consistency and accuracy.

### 2.4 Statistical analysis

Latent Growth Mixed Modeling (LGMM) in Mplus 8.3 identified suicide risk trajectories, with model fit evaluated using AIC, BIC, and entropy. SPSS 26.0 conducted Pearson correlations, repeated-measures ANOVA, and logistic regression. Significance was set at *α* = 0.05.

## 3 Results

### 3.1 Suicide risk scores across time points

Suicide risk scores at the three time points are presented in Table 1. Scores at the first measurement were significantly positively correlated with depressive symptoms scores (*p* < 0.001), and scores at the second and third measurements were also significantly correlated (*p* < 0.001). Repeated-measures ANOVA revealed a significant main effect of time on suicide risk (*F* = 110.516, *p* < 0.001). Post-hoc Bonferroni tests indicated that suicide risk scores decreased significantly from freshman to sophomore year and from sophomore to junior year (*p* < 0.001), suggesting a gradual decline in suicide risk over time.

**Table 1:** Descriptive statistics and correlation analysis at each measurement time point.

Variables	^−^x ± s	1	2	3	4
1. Suicide Behavior Risk at T1	4.51 ± 2.14	1.000			
2. Suicide Behavior Risk at T2	4.04 ± 1.77	−0.011	1.000		
3. Suicide Behavior Risk at T3	3.95 ± 1.70	−0.014	0.535**	1.000	
4. Depressive Emotion Score	44.58 ± 7.96	0.342**	0.013	0.024	1.000

**indicates *p* < 0.01. T1 = the first measurement; T2 = the second measurement; T3 = the third measurement.

### 3.2 Model fitting information

Goodness-of-fit analyses were conducted for LGMM models with 1 to 5 potential classes. As shown in Table 2, the 3-class model demonstrated the best fit, with lower values for LOG(L), AIC, BIC, and aBIC compared to models with fewer classes. The entropy value of 0.945 indicated high classification accuracy. The average probability matrix for class membership (Table 3) revealed that the probability of correct classification ranged from 90 to 97.8% across the three classes. According to the GRoLTS (Guidelines for Reporting on Latent Trajectory Studies) proposed by Van de Schoot et al. (2017), model selection requires comprehensive consideration of multiple factors. In this study, the three-trajectory model was chosen mainly for the following reasons: From the perspectives of theory and practical application, it can clearly distinguish student groups with different trends of suicide risk changes, providing a clear direction for colleges and universities to develop targeted intervention measures. A complex trajectory division is not conducive to practical operation. Although the AIC/BIC values of the four-trajectory and five-trajectory models are lower, in the three-trajectory model of this study, the “rapidly increasing suicide risk group” accounts for 3.2%, which already represents a high-risk subgroup. Moreover, this proportion is not significantly different from the lowest proportion of 1.9% in the four-trajectory model. At the same time, the three-trajectory model is more in line with the principle of model simplicity, facilitating the understanding and interpretation of results, and is conducive to the application of research findings in the field of college students’ mental health. When comparing models, we referred to multiple tools. The entropy value of the three-trajectory model is 0.945, indicating a relatively high classification accuracy. Considering these factors comprehensively, the three-trajectory model is the most suitable for this study.

**Table 2:** Model fitting results of latent class analysis for the developmental trajectories of suicidal behavior risk among college students.

Model	K	LOG(L)	AIC	BIC	aBIC	Entropy	LMR	BLRT	Class probabilities
1	8	−22176.587	44369.173	44418.951	44393.531	-	-	-	1
2	11	−21283.587	42589.174	42657.619	42622.666	0.953	0.0001	0.0001	0.917/0.083
3	14	−20646.621	41321.243	41408.355	41363.87	0.945	0.0036	0.0001	0.811/0.157/0.032
4	17	−20199.737	40433.473	40539.252	40485.234	0.986	0.0014	0.0001	0.143/0.774/0.063/0.019
5	20	−19873.973	39787.946	39912.392	39848.841	0.994	0.0018	0.0001	0.143/0.006/0.026/0.774/0.051

“-” indicates that the results of the latent class model fitting are not classified, and Entropy, LMR, and BLRT are not applicable; the number of freely estimated parameters; Log, likelihood test; AIC, Akaike Information Criterion; aBIC, adjusted BIC; Entropy, entropy value; LMR, likelihood ratio test; BLRT, Bootstrap - based likelihood ratio test; C, the number of latent classes.

**Table 3:** Average membership probabilities of each latent class.

Latent class	C1	C2	C3
C1	0.977	0.023	0.000
C2	0.099	0.900	0.001
C3	0.000	0.022	0.978

### 3.3 Developmental trajectories of suicide risk

**Figure 1:**
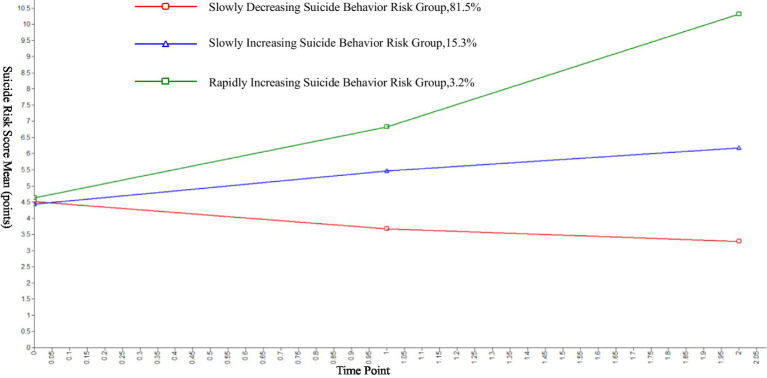
Estimated means and growth trajectories of various latent classes.

The LGMM analysis identified three distinct trajectories of suicide risk among college students (Figure 1).

C1: Slowly Decreasing Suicide Risk Group (3,020, 81.1%): Mean intercept: 4.358 (SE = 0.031, *t* = 141.182, *p* < 0.001); Mean slope: −0.536 (SE = 0.022, *t* = −24.276, *p* < 0.001); this group exhibited a gradual decline in suicide risk over time.

C2: Slowly Increasing Suicide Risk Group (586, 15.7%): Mean intercept: 4.540 (SE = 0.089, *t* = 50.805, *p* < 0.001); Mean slope: 0.816 (SE = 0.076, *t* = 10.748, *p* < 0.001); this group showed a gradual increase in suicide risk over time.

C3: Rapidly Increasing Suicide Risk Group (117, 3.2%): Mean intercept: 4.191 (SE = 0.259, *t* = 16.187, *p* < 0.001); Mean slope: 3.076 (SE = 0.158, *t* = 19.482, *p* < 0.001); this group demonstrated a sharp increase in suicide risk over time.

### 3.4 Univariate analysis of trajectory predictors

Univariate analyses revealed that gender, left-behind status, history of suicide among close relatives or acquaintances, positive psychological symptoms, and depressive symptoms were significantly associated with trajectory class membership (all *p* < 0.05; see Table 4).

**Table 4:** Univariate analysis of the latent classes of the developmental trajectories of suicide behavior risk among college students.

Variables	Total	Rapidly increasing suicide behavior risk group	Slowly increasing suicide behavior risk group	Slowly decreasing suicide behavior risk group	*X*^2^/*F* value	*p* value
Gender [*n* (Percentage, %)]						
Male	2,419 (65.0%)	64 (54.7%)	373 (63.7%)	1982 (65.6%)	6.445	**0.040**
Female	1,304 (35.0%)	53 (45.3%)	213 (36.3%)	1,038 (34.4%)		
Sexual Orientation [*n* (Percentage, %)]						
Heterosexual	3,408 (91.5%)	107 (91.5%)	532 (90.8%)	2,769 (91.7%)	0.519	0.772
Sexual minority	315 (8.5%)	10 (8.5%)	54 (9.2%)	251 (8.3%)		
Left - behind Experience [*n* (Percentage, %)]						
Left - behind	3,039 (81.6%)	84 (71.8%)	478 (81.6%)	2,477 (82.0%)	7.845	**0.020**
Non - left - behind	684 (18.4%)	33 (28.2%)	108 (18.4%)	543 (18.0%)		
Only Child [*n* (Percentage, %)]						
Only Child	834 (22.4%)	28 (23.9%)	125 (21.3%)	681 (22.5%)	0.582	0.748
Non - only child	2,889 (77.6%)	89 (76.1%)	461 (78.7%)	2,339 (77.5%)		
History of mental illness diagnosis [*n* (Percentage, %)]						
With a history of mental illness diagnosis	613 (16.5%)	15 (12.8%)	88 (15.0%)	510 (16.9%)	2.415	0.299
Without a history of mental illness diagnosis	3,110 (83.5%)	102 (87.2%)	498 (85.0%)	2,510 (83.1%)		
History of suicide among close relatives or acquaintances [*n* (Percentage, %)]						
With a history of suicide among close relatives or acquaintances	360 (9.7%)	20 (17.1%)	53 (9.0%)	287 (9.5%)	7.741	**0.021**
Without a history of suicide among close relatives or acquaintances	3,363 (90.3%)	97 (82.9%)	533 (91.0%)	2,733 (90.5%)		
Positive psychological symptoms [*n* (Percentage, %)]						
Positive psychological symptoms	561 (15.1%)	28 (23.9%)	82 (14.0%)	451 (14.9%)	7.754	**0.021**
Negative psychological symptoms	3,162 (84.9%)	89 (76.1%)	504 (86.0%)	2,569 (85.1%)		
Depressive symptom scores [points, X̄ ± s]	44.58 ± 7.96	45.29 ± 7.99	45.24 ± 7.86	44.42 ± 7.98	3.025	**0.049**

Bold values indicate statistical significance, i.e., meeting at least *p* < 0.05.

### 3.5 Multivariate logistic regression analysis

Multivariate logistic regression was conducted to examine predictors of trajectory class membership, with the “slowly decreasing suicide risk group” as the reference. Categorical variables were coded as follows: male = 1, female = 2; left-behind = 1, non-left-behind = 2; history of suicide among close relatives or acquaintances = 1, no history = 2; positive psychological symptoms = 1, negative symptoms = 2; depressive symptoms distress = 1, no distress = 2.

The results (Table 5) indicated that female students were more likely to belong to the “rapidly increasing suicide risk group” compared to males. Students with a left-behind experience, a history of suicide among close relatives or acquaintances, and positive psychological symptoms also had a higher probability of belonging to the “rapidly increasing suicide risk group.” Additionally, students with depressive symptoms distress were more likely to belong to the “slowly increasing suicide risk group.”

**Table 5:** Multivariate logistic regression analysis of the latent classes of the developmental trajectories of suicide behavior risk among college students.

Items	Regression coefficient	Standard error	Wald *X*^2^ value	*p* value	OR value(95% CI)
Rapidly increasing suicide behavior risk group					
Constant	−4.331	0.396	119.82	0.001	-
Gender	0.425	0.197	4.67	**0.031**	**0.654 (0.445–0.961)**
Left - behind experience	0.566	0.217	6.821	**0.009**	**1.761 (1.152–2.692)**
History of suicide among close relatives or acquaintances	0.556	0.263	4.456	**0.035**	**1.743 (1.041–2.920)**
Positive psychological symptoms	0.633	0.242	6.818	**0.009**	**1.882 (1.171–3.026)**
Depressive emotional distress	0.167	0.270	0.382	0.537	0.846 (0.499–1.436)
Slowly increasing suicide behavior risk group					
Constant	−1.614	0.087	344.969	0.001	-
Gender	−0.063	0.096	0.435	0.51	0.939 (0.777–1.133)
Left - behind experience	0.041	0.119	0.118	0.731	1.042 (0.825–1.315)
History of suicide among close relatives or acquaintances	−0.133	0.163	0.67	0.413	0.875 (0.636–1.204)
Positive psychological symptoms [*n* (Percentage, %)]	−0.150	0.141	1.13	0.288	0.861 (0.652–1.135)
Depressive emotional distress	0.258	0.124	4.358	**0.037**	**1.295 (1.016–1.650)**

“-” indicates the baseline value, which does not involve the influence of independent variables on the dependent variable, and there is no OR value. Bold values indicate statistical significance, i.e., confidence intervals do not include 1.

## 4 Discussion

This study revealed a gradual decline in suicide risk among college students over time, with the highest risk observed during the freshman year. This finding contrasts with Zhang and Chen (2021), who reported an initial increase followed by a decrease in suicidal ideation over 4 years. The discrepancy may be attributed to differences in study design and measurement tools. Our study employed a comprehensive assessment of suicide risk, including suicidal ideation, threats, and future likelihood, which may provide a more nuanced understanding of suicide risk trajectories. Additionally, the shorter time span (3 years vs. 4 years) and the use of average scores (vs. detection rates) in our study may have influenced the observed trends.

The LGMM analysis identified three distinct trajectories of suicide risk: a “slowly decreasing suicide risk group” (81.1%), a “slowly increasing suicide risk group” (15.7%), and a “rapidly increasing suicide risk group” (3.2%). The “slowly decreasing suicide risk group” accounted for the majority of the sample, driving the overall declining trend in suicide risk. However, the presence of a “slowly increasing suicide risk group” highlights the need for targeted interventions. Depressive symptoms distress was a significant predictor of membership in this group, consistent with previous research showing that depression mediates the relationship between stress and suicidal ideation. College students face numerous stressors, including academic pressures, interpersonal challenges, and career uncertainties, which can lead to chronic depression if not adequately addressed (Liu et al., 2024). The absence of effective coping mechanisms and external support may exacerbate these issues, increasing suicide risk over time. Early identification and intervention for students experiencing depressive symptoms are critical to preventing suicidal behavior. Therefore, colleges and universities should take a series of measures. They can further optimize mental health education courses, strengthen the education of depressive knowledge, and incorporate coping skill training. A regular screening mechanism for depressive symptoms should be established to promptly identify students in a depressive state or with depressive tendencies, especially those with potential suicide risks, and include them in the key attention list. For students with potential problems identified through screening, in-depth personalized risk assessments should be carried out. Finally, personalized intervention plans should be developed for each student based on the assessment results to ensure the pertinence and effectiveness of the intervention measures.

The “rapidly increasing suicide risk group” (3.2%) represents a critical subgroup requiring immediate attention. Female students, those with a left-behind experience, a history of suicide among close relatives or acquaintances, and positive psychological symptoms were more likely to belong to this group.

These findings align with Chen et al. (2024), who identified female gender and left-behind status as risk factors for suicidal ideation in early adolescence. In terms of social roles and expectations, society has specific expectations regarding women’s appearance and behavioral norms. Female students need to constantly adapt to these expectations during their growth process. For instance, the appearance anxiety caused by social media adds to their psychological burden. During the crucial growth stage, children with a left-behind experience lack continuous and stable emotional support and companionship from their parents, making it difficult for them to establish a strong sense of belonging in the family. This lack of belonging also extends to the school and social environments. When individuals are in a state of lacking belonging for a long time, they are prone to feelings of loneliness, helplessness, and abandonment. These negative emotions gradually erode their psychological defenses, increasing the risk of suicide. When left-behind students are emotionally distressed due to difficulties in life, which affects their studies and interactions with others, they may over-blame themselves and feel that they are a burden to others. This perception of being a burden further weakens their self-worth, making them feel that they have no positive contribution in social relationships, thus increasing the likelihood of suicide. Therefore, it is necessary to pay close attention to these high-risk student groups. For female students, in addition to regular mental health education courses, special female psychological growth courses should be offered. These courses can help female students better master effective emotion regulation methods and enhance their ability to cope with social stress. At the same time, schools can organize female mental health support groups, allowing female students to share their experiences and feelings in an atmosphere of mutual communication and support, and strengthening their psychological resilience. For students with a left-behind experience, “Growth Support Groups for Left-Behind Students” and other group counseling activities can be organized. Under the guidance of professional psychological teachers, group members can share their experiences, support each other, learn interpersonal skills in group interactions, enhance their sense of belonging and self-worth, improve their self-confidence, and thus improve their mental state.

Additionally, exposure to suicidal behavior has been shown to increase suicide risk through imitation (You et al., 2022). From the perspective of trauma psychology, the suicide of close relatives or acquaintances is a serious source of psychological trauma for students. This trauma may lead to symptoms related to post-traumatic stress disorder, such as repeated recall of the suicide event, nightmares, and avoidance of suicide-related scenes. These symptoms further affect their mental health and increase the risk of suicide. Reducing exposure to suicide-related information and providing timely psychological support are essential for mitigating this risk. Schools should prioritize early identification and intervention for high-risk students to prevent potential crises. Cognitive behavioral therapy, such as exposure therapy, can be used to help students gradually face the memories and emotions related to family suicide events in a safe and controlled environment, helping them reconstruct their perception of the event and reduce the negative impact of the trauma.

In conclusion, this longitudinal study identified three distinct trajectories of suicide risk among college students, with an overall declining trend over time. The findings highlight the importance of demographic, psychological, and social factors in shaping these trajectories, providing valuable insights for targeted prevention and intervention strategies. However, this study has several limitations. First, the sample was limited to freshmen from a single university in Guangdong Province, which may restrict the generalizability of the results. Second, while risk factors were extensively examined, protective factors such as psychological resilience (Feng et al., 2024; Wang et al., 2024), social connections (Cecchin et al., 2024; Meng and Xu, 2023), and gratitude traits (Xue et al., 2023) were not explored. Future research should investigate the role of these protective factors to provide a more comprehensive understanding of suicide risk and inform effective mental health interventions.

## Data Availability

The raw data supporting the conclusions of this article will be made available by the authors, without undue reservation.

## References

[ref1] Altavini C. S. Asciutti A. P. R. Santana G. L. Solis A. C. O. Andrade L. H. Oliveira L. G.. (2023). Suicide ideation among Brazilian college students: relationship with academic factors, mental health, and sexual abuse. J. Affect. Disord. 329, 324–334. doi: 10.1016/j.jad.2023.02.112, PMID: 36849006

[ref2] Cecchin H. F. G. da Costa H. E. R. Pacheco G. R. de Valencia G. B. Murta S. G. (2024). A mixed methods study of suicide protective factors in college students. Psicologia: Reflexão e Critica 37:35. doi: 10.1186/s41155-024-00315-0, PMID: 39269564 PMC11399479

[ref3] Che H. Y. Jia J. X. (2023). Meta-analysis of the detection rate of suicidal ideation and related factors among college students. Psychol Month 18, 69–71 + 131. doi: 10.19738/j.cnki.psy.2023.15.018

[ref4] Chen Z. Y. Chen Z. Y. Ouyang X. Y. Zhang Z. Liu M. Liu Z. (2023). One - year follow - up study on the outcome of suicide risk and its influencing factors among college students with positive psychological symptoms. Psychology 18, 67–70 + 105. doi: 10.19738/j.cnki.psy.2023.19.019

[ref5] Chen Z. Y. Zhang T. C. Zhang F. L. Zhou X. Wang A. Guo S.. (2024). Developmental trajectories of suicidal ideation in early adolescence: an analysis based on the latent growth mixture model. Modern Prev Med 51, 3314–3319. doi: 10.20043/j.cnki.MPM.202406431

[ref6] China Employment Training Technical Instruction Center (2005). Psychologist, Level 3. Beijing: Ethnic Publishing House.

[ref8] Feng H. Y. Cui H. B. Zhao H. P. Qiu M. (2024). A 30 - year review of research on suicidal ideation among Chinese college students - a visual analysis based on CiteSpace. Campus life Mental Health 22:395-400 + 461-462. doi: 10.19521/j.cnki.1673-1662.2024.05.005

[ref9] He Z. Q. Sun X. Y. Wang L. Q. Ding W. Q. Wang Z. Z. (2019). Correlation between social support, negative emotions, and suicidal behavior among college students. Chin. J. School Health 40, 704–706. doi: 10.16835/j.cnki.1000-9817.2019.05.017

[ref10] Li C. M. Li X. Y. Hao D. L. Duan H. Zhong X. Li Q. (2022). Current situation and influencing factors of suicidal ideation among college students. Psychol Month 17, 18–21. doi: 10.19738/j.cnki.psy.2022.18.006

[ref11] Liu Y. X. Zhang S. Yang L. Wang L. (2024). Exploration of short - term dynamic changes in suicidal ideation and its proximal risk factors among college students: based on the interpersonal theory of suicide. Chin. J. Clin. Psych. 32, 32–38. doi: 10.16128/j.cnki.1005-3611.2024.01.006

[ref12] Lu J. L. (2021). Self - loathing and suicidal behavior among college students: the chain mediating role of social connection and the sense of meaning in life. Huazhong Normal Univ. doi: 10.27159/d.cnki.ghzsu.2021.002310

[ref13] Meng Y. F. Xu S. M. (2023). Protective factors that block the evolution of suicidal ideation into suicide attempts among college students: composition, dimensions, and characteristics [C]//Chinese psychological society. Abstracts of the 25th National Congress of psychology in China-symposiums. Fujian Normal University School of Psychology; Fuzhou University Psychology Center: 2

[ref14] Qi X. Y. Zhang F. Q. Wang Y. K. (2024). Investigation and analysis of the potential suicide risk of vocational college students: taking Baoji vocational and technical college as an example. Psychol Month 19, 192–194. doi: 10.19738/j.cnki.psy.2024.01.059

[ref16] Shi X. L. Zhu Y. Dong J. X. Wang S. Xu L. Cai Y.. (2021). Reliability and validity of the suicidal behaviors questionnaire - revised (SBQ - R) in Chinese college students. Chinese J Health Psychol 29, 593–597. doi: 10.13342/j.cnki.cjhp.2021.04.025

[ref17] Sui J. (2024). Parenting styles and suicidal behaviors among college students: examining the mediating roles of coping, self - esteem, and depression. Behav Sci 14:666. doi: 10.3390/bs14080666, PMID: 39199062 PMC11351271

[ref18] Van de Schoot R. Sijbrandij M. Winter S. D. Depaoli S. Vermunt J. K. (2017). The GRoLTS-checklist: guidelines for reporting on latent trajectory studies. Struct. Equ. Model. Multidiscip. J. 24, 451–467. doi: 10.1080/10705511.2016.1247646

[ref19] Wang Z. Wang X. Peng Y. Liu C. He J. (2022). Recalled childhood maltreatment and suicide risk in Chinese college students: the mediating role of Psychache and the moderating role of meaning in life. J. Adult Dev. 30, 156–165. doi: 10.1007/s10804-022-09422-7, PMID: 40357353

[ref20] Wang Y. L. Wang D. Y. Shu Z. Y. He J. (2024). The impact of parental harsh parenting on suicidal ideation in early adolescence: the longitudinal moderating role of psychological resilience. Chin. J. Clin. Psych. 32, 549–554. doi: 10.16128/j.cnki.1005-3611.2024.03.011

[ref21] World Health Organization. (2019). World health statistics 2019: monitoring health for the SDGs (WHO World Health Statistics). Geneva: WHO.

[ref24] Xue Z. X. Ren Z. Y. Jing L. Li H. (2024). Classification decision tree analysis of influencing factors of suicidal behavior among college students. Psychol. Dev. Educ. 40, 421–430. doi: 10.16187/j.cnki.issn1001-4918.2024.03.13

[ref25] Xue Z. X. Zhang Y. Y. Xing Q. L. Li H. Jing L. (2023). Proximal and distal influencing factors in the transition from suicidal ideation to attempted suicide. Chin. J. Clin. Psych. 31, 542–548. doi: 10.16128/j.cnki.1005-3611.2023.03.008

[ref27] You J. Liu H. Liu X. C. Zhai D. Yang L. (2022). Analysis of influencing factors of suicidal ideation and suicide attempts among college students - based on the integrated motivational - volitional model of suicidal behavior. Chin. J. Clin. Psych. 30, 944–948. doi: 10.16128/j.cnki.1005-3611.2022.04.037

[ref28] Zhang J. J. Chen H. (2021). A 4- year follow - up study on suicidal ideation among college students in a certain university. Chin. J. School Health 42, 1524–1526. doi: 10.16835/j.cnki.1000-9817.2021.10.019

[ref30] Zhou Y. Liu J. D. Fan H. X. (2021). “A study on suicide risk from the perspective of the three-stage theory of suicide: a latent profile analysis based on chinese college students,” in Proceedings of the 23rd National Conference of the Chinese Psychological Society (Volume Upper). (pp. 740–741). School of Education Sciences, Shanxi University. doi: 10.26914/c.cnkihy.2021.042468

[ref31] Zhu J. Xie P. Zhang X. (2024). Social exclusion and suicide intention in Chinese college students: a moderated mediation model. Front. Psychol. 15. doi: 10.3389/fpsyg.2024.1354820, PMID: 38371706 PMC10869458

